# Comparative analysis of transvaginal sonographic cervical assessment and Bishop Score in predicting labour induction success at a tertiary hospital in Ghana

**DOI:** 10.4314/gmj.v60i1.3

**Published:** 2026-03

**Authors:** Teresa A Mensah, Kwaku Asah-Opoku, Kwame Adu-Bonsaffoh, Alim Swarray-Deen, Evans K Agbeno, Samuel A Oppong

**Affiliations:** 1 Department of Obstetrics & Gynaecology, Korle Bu Teaching Hospital, Accra, Ghana; 2 Maternal Fetal Medicine Unit, Cape Coast Teaching Hospital, Cape Coast, Ghana; 3 Department of Obstetrics & Gynaecology, University of Ghana Medical School, Accra, Ghana; 4 Department of Obstetrics & Gynaecology, University of Cape Coast, Cape Coast, Ghana

**Keywords:** Induction of labour, transvaginal sonographic cervical length, posterior cervical angle, Bishop score, Ghana

## Abstract

**Objectives:**

To compare transvaginal sonographic cervical assessment and Bishop Score (BS) in predicting successful induction (vaginal delivery within 24 hours) among low-risk postdate pregnancies (late term and post-term) induced at a tertiary hospital in Ghana.

**Design:**

An analytical cross-sectional study conducted between 2022 and 2023.

**Participants:**

Women with low-risk post-date pregnancies admitted for induction of labour. Transvaginal sonographic cervical assessment and BS were done for participants before the start of induction.

**Main outcome measures:**

The predictive abilities of the transvaginal sonographic cervical length (TVSCL), the BS and the posterior cervical angle (PCA) in predicting vaginal delivery.

**Results:**

Of 184 women recruited, 168 were included in the final analysis. The rate of vaginal delivery was 82.1%. Successful induction occurred in 117/168 participants (69.9%) [CI:62.1-76.5]. TVSCL ≤ 2.5cm was comparable in accuracy with BS ≥ 4 in predicting vaginal delivery. For predicting vaginal delivery within 24 hours, the accuracy of the PCA at ≥ 106 was marginally higher than the BS [AUC: 62.3; 61.0], although both were statistically significant. 95% of women preferred transvaginal sonographic assessment.

**Conclusion:**

BS and transvaginal sonographic assessment were comparable in predicting delivery when used for pre-induction cervical assessment. Transvaginal sonographic cervical assessment was better tolerated.

**Funding:**

None declared

## Introduction

Globally, the incidence of induction of labour has risen greatly over the past decade and is recognised as one of the strategies employed to reduce primary caesarean section rates, especially among pregnancies beyond 41 weeks+0 days.^1^ Worldwide, about 1 in 4 pregnant women in developed countries will have an induction of labour. 2 Rates vary by location and institution, with reportedly higher rates in developed countries; 20% in the United Kingdom and 4.4% in Africa.^3^ At Korle-Bu Teaching Hospital (KBTH), the average monthly induction rate is 13% according to the monthly maternal morbidity and mortality statistics presented in the year 2021.

Favourability of the cervix at the onset of induction is the most important marker of success. This has been traditionally assessed using the Bishop Score (BS).^4^ Transvaginal sonographic cervical length (TVSCL) assessment is also an acceptable tool for predicting success; a more common trend in developed countries.^5^ However, it is not widely used in developing countries currently because of the unavailability of equipment and skills.^6^

The BS is currently the most widely used pre-induction cervical assessment tool. It is considered a low-cost tool and can be used in low-resource settings where transvaginal ultrasound probes and provider skills may be unavailable.^7^ It is, however, fraught with several limitations. It is subjective, evidenced by its low reproducibility^7^,^8^, and significant inter- and intra-observer variability with low agreement.^7^,^9^

Digital examination cannot assess the supravaginal cervix, which usually accounts for about 50% of the cervical length.^4^ It is painful, 10 and lastly, it can only be audited by repeating a digital examination. The implication of these limitations is that patients may be inappropriately selected either for induction or caesarean section based on their subjective findings, with their associated complications and risks.

Transvaginal sonography (TVS) has several advantages. It can assess the entire cervical length without invading the endocervical canal.^11^ It is less painful^8^,^10^,^12^ and has the ability to assess other cervical parameters like the posterior cervical angle (PCA), funnelling and the cervical configuration. TVSCL is the most studied sonographic cervical parameter and can predict the likelihood of vaginal delivery or caesarean section, as well as the induction-to-delivery interval.^5^ It is an objective test with high interobserver agreement reported in the literature.^13^,^14^ Different cut-off values have been suggested to predict successful induction, with TVSCL values ranging from 1.8cm to 3.0cm.^10^,^15^,^16^

PCA is another cervical parameter identified as a predictor of successful induction 17,18 and provides a measure of cervical position.^17^ Values ranging from 99° to 120° have been identified as cut-off for predicting success.^17^–^19^ Transvaginal scans, although useful, require skill, are available only with probes, and come at an added cost.

Although studies have compared BS and TVSCL 4,10,15,20,21, most have been conducted in developed countries. Results from these studies have also been inconclusive, with inconsistent cut-off values. Whilst some studies demonstrated that the TVSCL was superior to the BS^10^,^15^, others showed the BS to be superior to the TVSCL^21^ and others demonstrated the TVSCL to be a significant predictor but not superior to the BS.^4^,^20^ A drawback to the comparisons is the observation that these studies used different study populations^15^,^21^,^22^ and different induction methods, agents and doses.^10^, ^21^, ^23^, ^24^ All of these heterogeneous variables could have impacted the success of labour induction. The study sought to determine whether, with a more homogeneous group and a single induction agent, we could identify a superior tool for predicting success.

The aim of this study was to compare transvaginal sonographic cervical assessment and BS in predicting successful labour induction among women with low-risk postdate pregnancies (between 41 and 43 weeks).

## Methods

### Study design and site

This was an analytical cross-sectional study, conducted between 21^st^ July 2022 and 30^th^ April 2023 at the Maternity unit of the KBTH, Accra. An average of 75 patients are induced per month according to the monthly maternal morbidity and mortality statistics. The common indications for induction of labour at KBTH over the past six months in decreasing order of frequency are postdate, hypertensive disorders, gestational diabetes, premature rupture of membranes and intrauterine foetal demise (IUFD). Postdate accounts for about 45% of induced cases, and low-risk postdate cases account for about 60% of those induced in a month.

Pre-induction cervical assessment is done using digital vaginal examination for all women, and the BS is determined and recorded on the morning of the induction of labour. Although the unit has a transvaginal probe and the requisite skill, a TVSCL assessment is not done.

### Study population

The study population was low-risk pregnant women admitted to the maternity unit of Korle Bu Teaching Hospital (KBTH) for a scheduled induction of labour on account of a late term (41weeks+0 days to 41weeks+6 days) or a post-term (42 weeks + 0 days to 43 weeks + 0 days) pregnancy. Low-risk was defined as a singleton, cephalic presentation, with no maternal medical condition (hypertension, diabetes, sickle cell disease, renal disease or human immunodeficiency virus infection); adequate pelvic capacity; no previous caesarean section and no foetal risk (intrauterine growth restriction or congenital anomalies). Excluded from the study were women with IUFD, allergy to misoprostol, abnormal Non-Stress Test (NST) or a history of a prior cervical surgery or insufficiency.

### Sample size calculation

Based on the study by Pandis et al.^10^ and using the sample size formula for determining adequate sensitivity or specificity for diagnostic tests as described by Jones et al.^25^, the sample size was calculated.

Sample size based on sensitivity: N(sN) = 158
Sample size based on specificity: N(sP) = 167

A sample size of 167 was estimated as the minimum required to achieve both sensitivity and specificity. After considering a 10% markup for loss to follow-up and incomplete data, we had a final sample size of N= 184.

### Sampling techniques

Pregnant women who met the inclusion criteria and consented to the study after counselling were recruited sequentially. The technique used was sequential recruitment.

### Study procedure

All participants had a transvaginal scan done for them on the morning of the induction. The principal investigator, a maternal-foetal medicine fellow, performed all transvaginal scans for the study. The BS was assessed by trained residents blinded to the sonographic findings within 30 to 60 minutes after the sonographic assessment. This time interval was chosen because it was assumed that the pain perception from one form of assessment would have waned. The patient's pain perception was assessed using a 10-point numerical pain rating scale and recorded after each assessment. Patients were then asked to choose their preferred method, if available, and to indicate the reason for their choice.

Misoprostol was given intravaginally in all patients and was inserted into the posterior fornix. The dosage used was 25 µg of misoprostol, administered every 6 hours for a maximum of 4 doses. This was aimed at standardising care and making the test easily reproducible. Induction of labour monitoring and labour management were undertaken as per the hospital's protocol, with no interference from the research team. All important labour events were recorded on the case report form by the research assistant. Pretraining over a two-day period was organised for all research assistants, and a pilot study to test the instruments and assess feasibility was done involving 10 patients (5% of the calculated sample size).

For the purpose of this study, 20 residents in Obstetrics were trained in BS assessment. Also, the principal investigator had a certificate of competence from the Fetal Medicine Foundation to perform TVSCL. Both measures aimed to improve the validity of results and minimise potential bias.

The primary outcome of the study was the predictive ability of the TVSCL, PCA, and BS for vaginal delivery. The secondary outcome was the pain scores post cervical assessment.

Definition of terms for the study:

- Successful induction of labour: vaginal delivery within 24 hours of onset of induction
- Failure of induction of labour: failure to have a vaginal delivery within 24 hours; that is, failure to deliver a patient vaginally in whom a safe vaginal delivery was initially expected. It therefore included:
  - Failure to induce effective uterine contractions within 24 hours^26^,^27^
  - Inability to achieve active labour^28^,^29^
  - Vaginal delivery after 24hrs of induction
  - The need to carry out a Caesarean section for failure to progress, foetal distress, infection, cord prolapse or Antepartum Haemorrhage (APH).^30^

### Measurement of cervical parameters with the transvaginal scan

The scan was performed using a 7 MHz transvaginal probe on a General Electric LOGIQ 5 Ultrasound machine. The technique used was as per the Fetal Medicine Foundation online cervical assessment training module.^5^ A sagittal plane of the cervix was obtained, and the endocervical mucosa was used as a guide to identify the internal and external os. The image was then magnified until the cervix occupied about 50% of the screen. The length of the cervix was measured as the linear distance between the “V-shaped” internal os and the “triangular-shaped” external os. Three measurements were taken and the shortest measurement was recorded. The PCA was then measured as the angle between the cervical canal and the posterior uterine wall. Also noted were the presence or absence of funnelling and the position of the cervix.

### Assessment of Bishop Score

The BS was assessed after the transvaginal scan. The parameters assessed included cervical length (not cervical effacement, as in the original BS), station, cervical dilatation, cervical consistency, and cervical position. The BS was determined, and the score was documented as in the KBTH induction of labour chart. The data for each participant were captured on the day of the induction of labour.

### Data management and analysis

Questionnaires and data collection sheets administered by trained research assistants were used to collect data. All the data captured were entered into Microsoft Excel (version 16) on a personal computer, which was password-protected. Data analyses were done using STATA 17. Descriptive statistics were presented in charts and tables. Categorical variables were expressed as frequencies, proportions and percentages. Receiver operating characteristic (ROC) curves were used to determine the optimal cut-off values of TVSCL, PCA, and BS for predicting successful induction (vaginal delivery within 24 hours) and vaginal delivery. The Area under the ROC curve (AUROC) was then calculated to assess the discriminatory ability of the tests at their optimal cut-off. Using the identified cut-off values, the data were then dichotomised into binary predictors. ROC curves were redrawn using the point of interest from the dichotomised data. Sensitivities, specificities, PPVs, and NPVs of the TVSCL, PCA, and BS for predicting the success of induction of labour (vaginal delivery within 24 hours of induction) and vaginal delivery were assessed from dichotomised data. In all statistical analyses, a p-value of ≤ 0.05 at a confidence interval of 95% was considered statistically significant.

### Ethical considerations

Approval for the study was obtained from the Korle-Bu Teaching Hospital Institutional Review Board, with reference number KBTH-STC/IRB/00036/2022, issued on 4^th^ July 2022. Informed written consent was obtained from all the study participants. No minors were included in the study. Participants were assured that declining to participate in the study would not affect the quality of care received, and their confidentiality was assured.

## Results

A total of 184 women were recruited for the study. Of these, 168 (91.3%) were included in the final analysis, with 16 cases excluded due to protocol deviation (14/16) and lost to follow-up (2/16). The mean age of participants was 29.2 ± 5.2 years. The sociodemographic and obstetric details of participants are shown in Table 1.

**Table 1:** Demographic Characteristics of participants

Characteristics	Frequency n (%)
**Age, in years**	
**< 35**	142 (84.5)
**≥ 35**	26 (15.5)
**Marital status**	
**Single**	54 (32.1)
**Married**	98 (58.3)
**Cohabiting**	16 (9.5)
**Level of education**	
**No formal education**	12 (7.1)
**Primary**	56 (33.3)
**Secondary**	53 (31.5)
**Tertiary**	47 (28.0)
**Occupation**	
**Professional**	45 (26.8)
**Trading**	51 (30.4)
**Artisan**	51 (30.4)
**Unemployed**	21 (12.5)
**Religion**	
**Christianity**	134 (79.8)
**Islam**	34 (20.2)
**BMI**	
**Underweight**	1 (0.6)
**Normal**	51 (30.4)
**Overweight**	54 (32.1)
**Obese I**	48 (28.6)
**Obese II**	14 (8.3)
**Parity**	
**Nulliparous (Para 0)**	68 (40.5)
**Primiparous (Para 1)**	46 (27.4)
**Multiparous (Para 2+)**	54 (32.1)
**Gestational age (In wks. + days)**	
**Late term (41+0 - 41+6)**	162 (96.4)
**Post term (42+0 – 43+0)**	6 (3.6)

### Preinduction cervical assessment

The findings on preinduction cervical assessment are shown in Table 2. The median BS was 5 (3.0 -6.0), the median TVSCL was 2.5 (1.9 -3.0), and the median PCA was 111 (97.5 - 125.5). For TVS assessment, only 16/168 participants (9.5%) had the presence of cervical funnelling, and for cervical position, 67.3% had a straight cervix and 32.7% a curved cervix.

**Table 2:** Pre-induction Cervical Assessment

Parameters	Range	Median (IQR)	Mean (SD)
**BS**	10.0	5 (3.0 -6.0)	4.85 (1.98)
**TVSCL**	4.78	2.5 (1.9 -3.0)	2.51 (0.91)
**Digital Cervical Length**	2.5	2.0 (1.0 -2.0)	1.88 (0.66)
**PCA**	110.0	111.0 (97.5 -125.5)	112.3 (20.73)

For the BS assessment, 41.7% had an unfavourable cervix (BS ≤ 4), 2.4% had a favourable cervix (BS ≥ 9), and 55.9% had a moderately favourable cervix (BS 5-8). The majority of participants had either a centrally (49.4%) or posteriorly (43.5%) positioned cervix, while 50.6% had a soft cervix and 8.3% had a firm cervix.

### Optimum cut-off value for transvaginal sonographic cervical length, posterior cervical angle and Bishop Score and their predictive values

**Figure 1:**
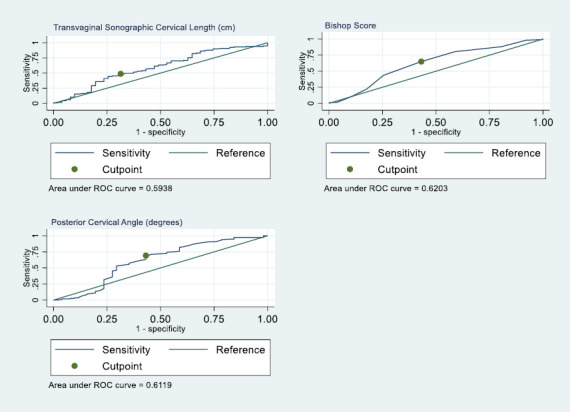
ROC curve for predicting successful induction of labour (TVSCL at 2.31cm, BS at 4 and PCA at 106°)

ROC curves constructed to determine the best cut-off values for TVSCL, BS and PCA in predicting successful induction of labour (vaginal delivery within 24 hours) are shown in Figure 1 with the AUROC curve. The TVSCL optimal cut-off value was 2.31cm, with a sensitivity of 49.0% and a specificity of 69.0%. The BS cut-off value was 4, with a sensitivity of 65.0% and a specificity of 57.0%. The PCA cut-off value was 106, with a sensitivity of 69.0% and a specificity of 57.0%.

**Figure 2:**
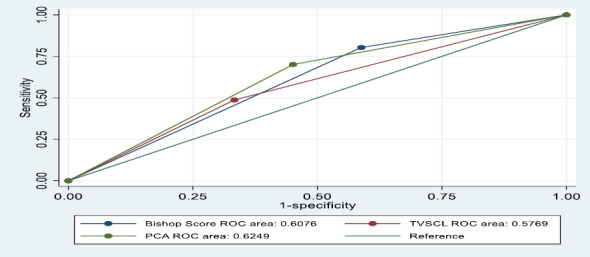
ROC curves for predicting successful induction of labour (DICHOTOMIZED: TVSCL, BS and PCA)

Using the identified cut-off values from the ROC curves, the data were dichotomised into binary predictors (TVSCL: ≤ 2.31cm and > 2.31cm; BS ≥ 4 and < 4; PCA: ≥106° and <106°) and new ROC curves drawn for the category of interest (TVSCL: 2.31cm; BS ≥ 4 and PCA: ≥ 106°). Table 3 illustrates the predictive values of the TVSCL, BS, and PCA (dichotomised), along with their statistical significance. Figure 2 shows the ROC curve for the dichotomised data for TVSCL, BS and PCA.

**Table 3:** Predictive values of TVSCL, BS and PCA (dichotomised) for Successful Induction of Labour (vaginal delivery within 24 hours)

	Cut-off	Sensitivity (%)	Specificity (%)	AUC (95% CI)	PPV (%)	NPV (%)	Std. Err	P-value
**TVSCL**	≤ 2.31	48.7	66.7	58.0 (49.7, 65.7)	77.0	36.2	0.283	**<0.001**
**BS**	≥ 4.0	80.3	41.2	61.0 (52.4, 71.6)	75.8	47.7	0.483	**<0.001**
**PCA**	≥106	70.1	54.9	62.3 (54.4,70.6)	78.1	44.4	4.001	**<0.001**

**Figure 3:**
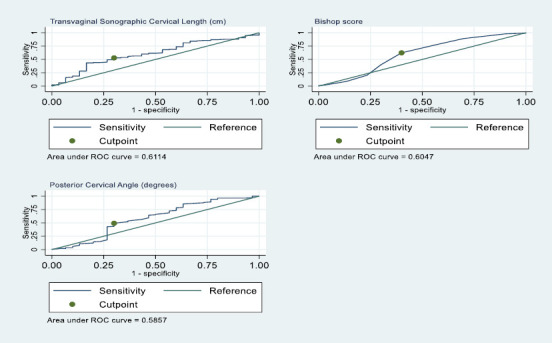
ROC curve for predicting vaginal delivery (TVSCL at 2.5cm, BS at 4 and PCA at 112°)

ROC curves were reconstructed to determine the best cutoff values for TVSCL, BS and PCA in predicting vaginal delivery. Figure 3 shows the AUROC curves for TVSCL, BS and PCA. The TVSCL optimal cut-off value was 2.5cm, with a sensitivity of 53.0% and a specificity of 70.0%. The BS cut-off value remained 4, with a sensitivity of 62.0% and a specificity of 60%. The PCA cut-off value was 112° with a sensitivity of 49.0% and a specificity of 70.0% at the cut-off value.

**Figure 4:**
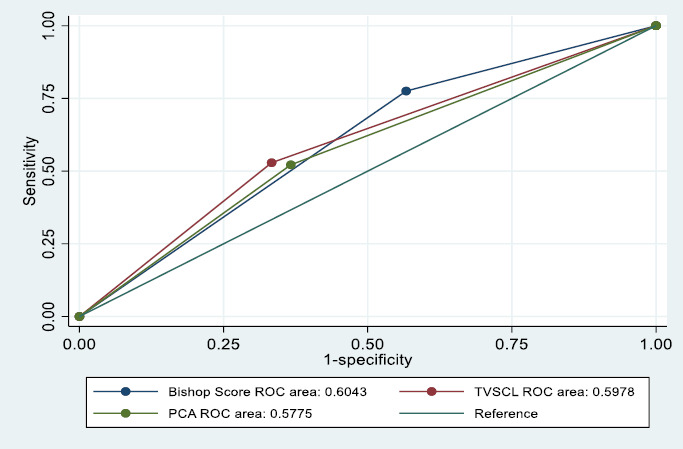
ROC curves for predicting vaginal delivery (Dichotomised: TVSCL, BS and PCA)

Using the identified cut-off values from the ROC curves, the data were dichotomised into binary predictors (TVSCL: ≤ 2.5cm and > 2.5cm; BS ≥ 4 and < 4; PCA ≥112° and <112°) and new ROC curves drawn for the category of interest (TVSCL ≤ 2.5cm; BS ≥ 4 and PCA ≥ 112°). Table 4 illustrates the predictive values of the TVSCL, BS, and PCA (dichotomised), along with their statistical significance. Figure 4 shows the ROC curve for the dichotomised data for TVSCL, BS and PCA.

**Table 4:** Predictive values of TVSCL and BS (dichotomised) for Vaginal delivery

	Cut-off	Sensitivity (%)	Specificity (%)	AUC (95% CI)	PPV (%)	NPV (%)	Std. Err	P-value
**TVSCL**	≤ 2.5	52.9	66.7	60.0 (52.0, 69.0)	88.0	23.5	0.300	**<0.001**
**BS**	≥ 4.0	77.5	43.3	60.0 (51.0, 70.0)	86.3	29.5	0.510	**<0.001**
**PCA**	≥112	52.2	63.3	58.0 (48.0,67.0)	86.7	22.4	6.294	**<0.001**

### Preference for cervical assessment methods

**Figure 5:**
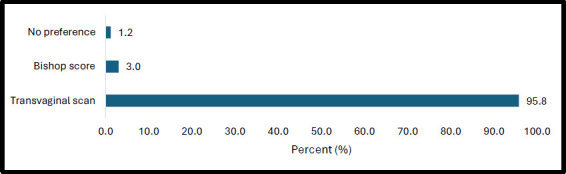
Participants' Preference for a Pre-induction Cervical Assessment Method

The majority of participants, 161/168 (95.8%), preferred the transvaginal sonographic cervical assessment. Figure 5 shows the breakdown of participants' preferences for cervical assessment methods.

The mean pain score post-digital assessment was 5.98 2.6, compared with a mean pain score post-TVS assessment of 0.13 ± 0.47. The maximum pain score for TVS assessment was 3, whereas the maximum for digital assessment was 10. Of the 161 women who preferred the TVS; 160/161 (99.4%) felt it was less painful with only one person reporting no difference in pain. Also, 120 of the 160 women (75%) felt the TVS was less invasive. Only 5/168 participants (3%) preferred the digital assessment. Although they felt it was more painful and invasive, 4 out of 5 thought it was more natural, and 1 thought it prepared her for the process of labour.

A linear regression analysis revealed a strong negative correlation between parity and the post-BS pain score. Parous women were less likely to experience a greater pain score. OR (95% CI) = 0.41 (0.18, 0.91); p = 0.029). (See Table 5). The difference in mean pain scores post-BS assessment between parous and nulliparous women was significant. (t (166) = 2.21), p = 0.029. Nulliparous women (Mean = 6.51, 2.46) compared with Parous women (Mean = 5.62, 2.66).

**Table 5:** Correlation between Maternal characteristics and post BS Pain Score

	Coefficient	Std. Err	t	P-value	95% CI
**Parity**					
**Nulliparous**	Ref				
**Parous**	-0.89	0.41	-2.21	**0.029**	-1.70, -0.09
**Constant**	6.51	0.31	20.83	**< 0.001**	5.90, 7.13
**Educational level**					
**No formal education**	Ref				
**Primary**	0.42	0.79	0.53	0.598	-1.14, 1.97
**Secondary**	2.13	0.79	2.69	**0.008**	0.57, 3.70
**Tertiary**	2.10	0.80	2.62	**0.010**	0.51, 3.58
**Constant**	4.58	0.72	6.40	**< 0.001**	3.17, 6.00
**Religion**					
**Christian**	Ref				
**Islam**	-0.72	0.50	-1.43	0.154	-1.70, 0.27
**Constant**	6.13	0.22	27.27	**< 0.001**	5.68, 6.57
**Marital status**					
**Single**	Ref				
**Married**	-0.10	0.44	- 0.23	0.817	- 0.97, 0.77
**Cohabiting**	-0.53	0.75	- 0.71	0.478	- 2.00, 0.94
**Constant**	6.09	0.36	17.08	**< 0.001**	5.39, 6.80

There was a strong positive correlation between educational level and post-BS pain score. Women with secondary or tertiary education were more likely to experience a greater pain score compared to women with no formal education.

OR (95% CI) for Secondary education:8.41 (1.76, 40.45), p=0.008.
Tertiary education: 8.16 (1.66, 35.87), p = 0.010).

Religion and marital status did not significantly affect the post-BS pain score. Although Muslims were less likely to experience a greater pain score compared to Christians, this was not statistically significant. Similarly, women who were married or cohabiting were less likely to experience a greater pain score compared to women who were single, but this was also not statistically significant. (See Table 5).

## Discussion

The study determined the cut-off values for the TVSCL, BS, and PCA; compared their predictive abilities; and assessed the acceptability of both pre-induction cervical assessment methods. The study identified cut-off values of 2.31cm for TVSCL, 4 for BS and 106° for PCA. At this cut-off, all 3 methods significantly predicted successful induction of labour (vaginal delivery within 24 hours). For predicting vaginal delivery, the optimum cut-off value for TVSCL was 2.5cm, 4 for BS and 112° for PCA. Similar cut-off values have been reported in other studies.^18^,^19^,^31^–^33^

The AUROC curves for TVSCL, BS and PCA in predicting successful induction of labour were 58, 61 and 62.3, respectively. The PCA had the highest accuracy, although all three were statistically significant. Also, the ROC curves for BS and TVSCL crossed, indicating that the two methods complement each other and that, in certain regions of the curve, one is preferred over the other. This was evidenced in the analysis, with TVSCL being more specific (66.7% vs. 41.2%), whilst BS was more sensitive (80.3% vs. 48.7%). For predicting vaginal delivery, both TVSCL and BS had similar accuracy, with both measures having an AUROC of 60, and both were statistically significant. The PCA had the lowest accuracy. TVSCL was still more specific (66.7% vs. 43.3%), and BS was more sensitive (77.5% vs. 52.9%).

TVS was well tolerated by the participants. A total of 95.8% of participants preferred the TVS to the BS. Of these, 99.4% reported less pain during TVS assessment. About 75% of those who preferred the TVS also reported that the procedure was less invasive than the BS. These findings are compatible with those of previous studies.^32^,^34^,^35^

Gunes et al showed a negative correlation between the number of live births a woman had previously had and the level of discomfort from a digital vaginal assessment.^36^ In the current study, a similar association was observed, with a strong negative correlation between parity and post-BS pain. The mean post-BS pain score in nulliparous women was 6.51, which was significantly higher than that in the parous group, 5.62, p=0.029. The level of education had a significant effect on the post-BS pain score. Women with secondary and tertiary education had a greater likelihood of experiencing more pain compared to women with no formal education. Religion and marital status, however, did not have a significant effect on the post-BS pain score in the current study. Several factors have been identified in previous studies to predict the willingness of women to have a transvaginal scan done for them.^34^,^37^ These include the woman's parity, ethnicity, prior painful vaginal examination and educational level.^34^,^35^,^37^,^38^ Whilst Okeji et al. identified that higher educational level increases the acceptability of transvaginal scans 35, Komolafe and colleagues identified that secondary education, rather than tertiary education, was associated with a higher acceptability.^38^ Similarly, studies have identified no significant association between religion^37^,^39^ or marital status 39 and the willingness to have a transvaginal scan.

### Consideration for implementation/practice implications

An ideal screening test should correctly identify all patients with and without the outcome. In reality, the majority of tests will, however, fall short of being ideal. The current findings of the study suggest that over 95% of women tolerated the TVS and preferred it to the BS, mainly because it was less painful and also because it was less invasive.

Having a test which is extremely more comfortable, with similar accuracy to the current standard (BS), may be more desirable as a first-line screening tool for predicting successful induction of labour and vaginal delivery. Using TVS (PCA or TVSCL) as the first-line screening tool for low-risk postdate pregnant women, at a cut-off of 106 or 2.5cm, will predict 78% - 88% of those who will have a successful induction or vaginal delivery. These patients will then not be subjected to the painful BS assessment before having induction of labour. Patients with TVSCL >2.5cm will then undergo digital assessment for BS, which, as suggested by this study, is a more sensitive test. The use of the BS as the second screening test will identify true positives among those with TVSCL >2.5cm, thereby complementing TVSCL's low sensitivity in predicting low-risk patients.

This stepwise screening tool (which we will call “T-MENS” stepwise induction of labour screening tool) will, in effect, save a number of women from undergoing the more painful and uncomfortable digital assessment for BS prior to the start of induction. Both tests will complement each other if used as a stepwise screening tool. Fewer women with a high risk of having a failed induction will have induction started if this stepwise approach is done, and so the success rates of induction of labour will also be improved, and the complications of failure will be reduced.

Transvaginal scans may not be readily available in all hospitals in Ghana. However, in a tertiary setting like KBTH, where TVS is available, and the expertise to perform TVS (TVSCL and PCA) exists, performing TVS (TVSCL and PCA) as the first screening tool may be useful for counselling patients on their probability of a successful induction of labour, whilst subjecting them to less pain. A subsequent stepwise screening with the BS may improve success rates of vaginal delivery in low-risk postdate pregnancies, hence decrease the complications of failed induction of labour.

The study's strengths include having a single operator perform all transvaginal scans, thereby eliminating interobserver variability. A single induction agent and protocol were used, thus standardising the induction process. Heterogeneity was also minimised as only low-risk postdate pregnant women were recruited for induction in the study. Lastly, whilst previous studies have concentrated on the predictive value of a threshold score, this study has sought to dichotomise the data to make it more user-friendly for the clinician and patient, and more practical for clinical work.

The study is limited by the use of a single tertiary centre for participant recruitment, and thus, the findings may not be representative of the country. KBTH, however, is the biggest referral facility in Ghana, with attendants coming from a wide catchment area. Cost implications were not factored in the acceptability of the transvaginal sonographic assessment. Lastly, the use of predictive values for binary predictors can lead to some loss of detail at the point of interest and to a possible underestimation of the AUROC. It is advised that the interpretation given to the results is based on the inherent limitations of using binary predictors.

The study identified cut-off values for TVSCL, PCA, and BS that will be clinically useful for predicting successful induction of labour and vaginal delivery among Ghanaian women with low-risk postdate pregnancies (between 41 and 43 weeks) assessed for induction. It also introduces the “T-MENS” stepwise induction of labour screening tool to enhance patient satisfaction, improve the induction experience, and facilitate appropriate patient selection for induction of labour.

We recommend that transvaginal sonographic cervical assessment be integrated into the pre-induction cervical assessment of postdate pregnancies at the maternity unit of KBTH and in other settings with similar circumstances (where expertise for and availability of transvaginal ultrasound exist), and be used as a guide for patient counselling and decision-making. It may be a viable or more desirable alternative to the BS for women who cannot tolerate the pain of digital assessment due to either a prior negative experience or personal preference, given its superior patient tolerability and comparability to the current standard for predicting outcome. A cost-effectiveness study is also recommended to further research its implications for implementation strategies.

## Conclusion

Both cervical assessment methods were comparable in their ability to predict successful induction of labour or vaginal delivery. Transvaginal sonographic cervical assessment, however, was better tolerated than the digital assessment for BS, mainly because it was less painful and also because it was less invasive.

## References

[1] (2014). Obstetric care consensus no. 1: Safe prevention of the primary caesarean delivery. Obstetrics and Gynaecology.

[2] World Health Organization (2018). WHO recommendations: Induction of labour at or beyond term.

[3] Bukola F, Idi N, Mmimunya M (2012). Unmet need for induction of labour in Africa: Secondary analysis from the 2004 - 2005 WHO Global Maternal and Perinatal Health Survey (A cross-sectional survey). BMC Public Health.

[4] Crane JMG (2006). Factors Predicting Labour Induction Success: A Critical Analysis. Clin Obstet Gynecol.

[5] Fetal Medicine Foundation (FMF) Cervical Assessment [Internet].

[6] Venkatayogi N, Gupta M, Gupta A (2023). From Seeing to Knowing with Artificial Intelligence: A Scoping Review of Point-of-Care Ultrasound in Low-Resource Settings. Applied Sciences.

[7] Mlodawski J, Mlodawska M, Plusajska J (2023). Repeatability and Reproducibility of Potential Ultrasonographic Bishop Score Parameters. J Clin Med.

[8] Bajpai N, Bhakta R, Kumar P (2015). Manipal cervical scoring system by transvaginal ultrasound in predicting successful labour induction. Journal of Clinical and Diagnostic Research.

[9] D'Souza RD (2019). Bishop Score as a Measurement Instrument [30H]. Obstetrics & Gynaecology.

[10] Pandis GK, Papageorghiou AT, Ramanathan VG (2001). Preinduction sonographic measurement of cervical length in the prediction of successful induction of labour. Ultrasound in Obstetrics and Gynaecology: The Official Journal of the International Society of Ultrasound in Obstetrics and Gynaecology.

[11] Park KH, Kim SN, Lee SY (2011). Comparison between sonographic cervical length and Bishop score in preinduction cervical assessment: A randomized trial. Ultrasound in Obstetrics and Gynaecology.

[12] Alvarez-Colomo C, Gobernado-Tejedor JA (2016). The validity of ultrasonography in predicting the outcomes of labour induction. Arch Gynecol Obstet.

[13] Gascón A, Goya M, Mendoza M (2020). Intraobserver and interobserver variability in first-trimester transvaginal ultrasound cervical length. Journal of Maternal-Fetal and Neonatal Medicine.

[14] Stein W, Hellmeyer L, Schmidt S (2011). Intraobserver and interobserver reliability of transvaginal cervical length measurements and quantitative ultrasound tissue characterization of the cervix in the second and third trimester of pregnancy. Ultraschall in der Medizin.

[15] Bastani P, Hamdi K, Abasalizadeh F (2011). Transvaginal ultrasonography compared with Bishop score for predicting caesarean section after induction of labor. Int J Womens Health.

[16] Alaa D, Salem NA, Abdel-Hak A (2022). Role of pre-induction transvaginal ultrasound measurement of cervical length in prediction of labour induction success. Medical Journal of Cairo University.

[17] Rane SM, Guirgis RR, Higgins B (2004). The value of ultrasound in the prediction of successful induction of labour. Ultrasound in Obstetrics and Gynaecology.

[18] Hassan SSM, Ahmed Omar AE, Essam AM (2021). Cervical length and posterior-cervical angle in prediction of successful induction of labour. Ginekologia Poloznictwo.

[19] Keepanasseril A, Suri V, Bagga R (2007). Pre-induction sonographic assessment of the cervix in the prediction of successful induction of labour in nulliparous women. Australian and New Zealand Journal of Obstetrics and Gynaecology.

[20] Ezebialu IU, Eke AC, Eleje GU (2015). Methods for assessing pre-induction cervical ripening. Cochrane Database of Systematic Reviews.

[21] Cengiz Huseyin, Yalvac Serdar, Yavuzcan Ali (2012). Prediction of successful induction of labour with dinoprostone in a homogenous group of patients. S Afr J Obstet Gynaecol.

[22] Ransiri PADL, Rathnayake RMCJ, Kandauda IC (2018). Comparison of transvaginal ultrasonographic and digital cervical assessment in predicting successful induction of labour in nulliparous pregnancy beyond 40 weeks with unfavourable cervix – A prospective cohort study. Sri Lanka Journal of Obstetrics and Gynaecology.

[23] Kanwar SN, Reena P, Priya BK (2015). A Comparative Study of Trans vaginal Sonography and Modified Bishop's Score for Cervical Assessment before Induction of Labour. Scholars Journal of Applied Medical Sciences.

[24] Li PC, Tsui WL, Ding DC (2023). The Association between Cervical Length and Successful Labour Induction: A Retrospective Cohort Study. Int J Environ Res Public Health.

[25] Jones SR, Carley S, Harrison M (2003). An introduction to power and sample size estimation. Emergency Medicine Journal.

[26] National Institute for Health and Care Excellence (2021). Inducing labour (update) NG207.

[27] Spong CY, Berghella V, Wenstrom KD (2012). Preventing the first caesarean delivery: Summary of a joint eunice kennedy shriver national institute of child health and human development, society for maternal-fetal medicine, and American College of Obstetricians and Gynaecologists Workshop. Obstetrics and Gynaecology.

[28] Caughey AB, Sundaram V, Kaimal AJ (2009). Systematic review: Elective induction of labour versus expectant management of pregnancy. Ann Intern Med.

[29] Banõs N, Migliorelli F, Posadas E (2015). Definition of Failed Induction of Labour and Its Predictive Factors: Two Unsolved Issues of an Everyday Clinical Situation. Fetal Diagn Ther.

[30] Frederiks F, Lee S, Dekker G (2012). Risk factors for failed induction in nulliparous women. Journal of Maternal-Fetal and Neonatal Medicine.

[31] Laencina AMG, Sánchez FG, Gimenez JH (2007). Comparison of ultrasonographic cervical length and the Bishop score in predicting successful labour induction. Acta Obstet Gynecol Scand.

[32] Abdullah ZHA, Chew KT (2022). Pre-induction cervical assessment using transvaginal ultrasound versus Bishops cervical scoring as predictors of successful induction of labour in term pregnancies: A hospital-based comparative clinical trial. PLoS One.

[33] Rane SM, Guirgis RR, Higgins B (2003). Pre-induction sonographic measurement of cervical length in prolonged pregnancy: The effect of parity in the prediction of the need for Caesarean section. Ultrasound in Obstetrics and Gynaecology.

[34] Clement S, Candy B, Heath V (2003). Transvaginal ultrasound in pregnancy: Its acceptability to women and maternal psychological morbidity. Ultrasound in Obstetrics and Gynaecology.

[35] Okeji MC, Agwuna KK, Ihudiebube-Splendor CN (2017). Transvaginal Sonography: Perception and attitude of Nigerian women. BMC Womens Health.

[36] Güneş G, Karaçam Z (2017). The feeling of discomfort during vaginal examination, history of abuse and sexual abuse and post-traumatic stress disorder in women. J Clin Nurs.

[37] Atalabi OM, Morhason-Bello IO, Adekanmi AJ (2011). Transvaginal ultrasonography: A survey of the acceptability and its predictors among a native African women population. Int J Womens Health.

[38] Komolafe JO, Akindele RA, Akinleye CA (2016). Awareness and acceptance of transvaginal ultrasound scanning among ever pregnant women in Nigeria. Women's Health and Gynaecology.

[39] Akinmoladun J, Oluwasola TAO (2017). Transvaginal ultrasound during pregnancy: Perception and acceptability of antenatal clinic attendees at the University College Hospital, Ibadan. Trop J Obstet Gynaecol.

